# Risk factors and birth prevalence of birth defects and inborn errors of metabolism in Al Ahsa, Saudi Arabia

**DOI:** 10.4314/pamj.v8i1.71064

**Published:** 2011-02-23

**Authors:** Waleed Hamad Al Bu Ali, Magdy Hassan Balaha, Mohammed Saleh Al Moghannum, Ibrahim Hashim

**Affiliations:** 1 Dammam University, Saudi Arabia; 2 King Faisal University, Saudi Arabia; 3 Al Ahsa Maternity Hospital, Saudi Arabia

**Keywords:** Birth defects, inborn errors of metabolism, newborn, Saudi Arabia

## Abstract

**Background**

Birth defects and inborn errors of metabolism are related to variable poor perinatal and neonatal outcomes. Our aim was to explore the pattern and prevalence of birth defects and metabolic birth errors in Al-Ahsa Governorate in the Eastern Province of Saudi Arabia.

**Methods**

This retrospective case control study was done from April 2006 to 2009. Children with any birth defect or metabolic errors of metabolism at birth or in the neonatology section were our sample for study. Control group was randomly selected from the cases with normal live births. Blood tests were performed for children suspected to suffer from genetic blood disorders. The principal BD as per the International Classification of Diseases-10 (ICD-10) code was also noted.

**Results**

Out of 38001 live births, birth defects were found in 1.14% and errors of metabolism were detected in 0.17%. The most common birth defects were craniofacial malformations. The 3-methylcrotonyl-CoA carboxylase deficiency was the most common inborn errors of metabolism. Consanguinity, rural residence and prematurity were associated with significant rise in birth defects. On the other hand, consanguinity and low birth weight were associated with significant rise in metabolic errors.

**Conclusion**

First cousins consanguinity represented the most significant risk factor for birth defects and inborn errors of metabolism. High degree of inbreeding, consanguinity may exacerbate underlying recessive genetic risk factors.

## Background

Birth defects are a major cause of perinatal and neonatal death [1]. Worldwide, the prevalence rates of all genetic birth defects combined range from a high of 82/1,000 live births in low-income regions to a low of 39.7/1,000 live births in high-income regions [2]. These malformations have multifactorial etiologies and 40% of cases are idiopathic but there is an impression that they are more prevalent in populations with consanguineous marriages [3].

Epidemiologic surveys of birth defects in various part of the world and among different ethnic groups with widely varying marital habits, socioeconomic status and environment not only help in understanding the frequency of malformations in specific areas but also contribute to the general knowledge about the predisposing factors and different patterns of birth defects. There may be regional variations in the rate and pattern of birth defects or these could vary over time [4]. Birth defects are associated with several adverse pregnancy outcomes, such as perinatal mortality, growth restriction, preterm delivery, breech presentation, preeclampsia, placental abruption and also distorted sex ratio [5]. Inborn errors of metabolism (IEM) comprise a large class of genetic diseases involving disorders of metabolism. The majority are due to defects of single genes that code for enzymes that facilitate conversion of various substances (substrates) into others (products). In a Western study, the overall incidence of the inborn errors of metabolism were estimated to be 70 per 100,000 live births or 1 in 1,400 births, overall representing more than approximately 15% of single gene disorders in the population [6].

The prevalence of hereditary blood disease has the highest incidence in Eastern province of KSA. Sickle cell disease is present throughout Saudi Arabia; particularly common in the eastern and southern provinces: Qatif (eastern region) 17.0 %, Gizan (southern region), 10.3%, Ula (Northern region) 8.1 % and Mecca (western region) 2.5 % [7] In Saudi Arabia the reported frequency of ß-thalassemia ranges from 1% in some areas to 5% in others. In Saudi Arabia the frequency of μ-thalassemia ranges from 12%-60% in different parts of the country with the highest frequency in the eastern province. [8] Despite it is a common disease, it is not part of the IEM.

In Saudi Arabia, a recent study estimated the incidence of major and minor birth defects among live born infants to be 27.1/1000 live births and the highest incidence was for cardiovascular (7.1/1000 live births), and musculoskeletal/limb malformations (4.1/1000 live births) [9]. Many studies found the incidence of congenital abnormalities to be 23/1000 live births. The incidence for birth defects of the gastrointestinal tract was 1.3 per 1000 live births, for neural tube defects (NTD) 1.9/1000 live births, and for Down’s syndrome 1.8 per 1000 live births [10-13].

To our knowledge there is no research on the pattern or prevalence of birth defects and metabolic birth errors the in Al Ahsa Governorate in the Eastern Province of Saudi Arabia. The aim of this research was to explore the pattern and birth prevalence of birth defects and metabolic errors of metabolism in Al Ahsa Governorate.

## Methods

This study was conducted in the Maternity Hospital in Al Ahsa, Eastern Region, Saudi Arabia; which is the largest province of Saudi Arabia. It has an area of 710,000 km² and a population of 4,105,780. Al-Ahsa City where we are working serves about 660,788 of population. This retrospective case control study was done by reviewing of Al Ahsa Maternity and Children hospital (MCH) files. It was done through revising a part of a clearly defined systemic registry sheet in the neonatology Department. All babies born from April 2006 to 2009 were our total study population. Children with any birth defect (BD) or metabolic errors of metabolism at birth or in the neonatology section were our sample for study. Control group was randomly selected from the cases with normal live births; as 4:1. Four normal cases were selected for each one case with either BDs or metabolic errors.

Major birth anomalies were detected by the attending Obstetrician and all anomalies and inborn errors of metabolism were detected by neonatology consultants using a fixed protocol by all of them. Birth defects are diagnosed at the time of delivery or during the following stay at neonatology unit or during early neonatal follow up period, traditionally around seven days. Those identified with anatomical deformities were registered after detailed clinical examination. Children underwent radiological (312 out of the diagnosed 426) and sonographic evaluation (all 426) to identify any other anomalies in addition to the principal defect. The metabolic error patients were evaluated on the basis of their alerting signs for common genetic metabolic diseases and/or family history of either unexplained neonatal death or previously affected members.

Only cases of recurrent anomalies were assessed through the Genetics consultant by this systematic approach of full pedigree. Cases with first time occurrence were just followed by simple family history.

As retrieved from the Laboratory data, the biochemical investigations included analysis of quantitative plasma and cerebrospinal fluid amino acids, urine organic acids, oligosaccharidoses, and glycosaminoglycans. For urea cycle disorders, diagnosis was based on the presence of hyperammonemia and the typical plasma amino acids profiles. The diagnosis was confirmed by enzyme activity estimation in the blood of all patients with fatty acid oxidation disorders. Biotinidase deficiency was diagnosed by blood enzyme assay. The cases of propionic aciduria, methylmalonic aciduria, and maple syrup urine disease (MSUD) had their diagnoses confirmed by enzyme assay. The diagnosis of all glycogen storage disorders was confirmed by enzyme activity estimation on cultured leukocytes.

These are the standardized screening tests in all metabolic disorders, which may need confirmation by tissue culture and Tandem mass spectrometry. All the confirmatory tests were done and the cases are managed in a tertiary center in Riyad. All the cases were managed with nutrition care from the first day. Specific therapies, milk formula and avoiding some food were advised based on the tertiary center care.

It is worth mentioning that the detection of all blood disorders is mandatory for all newborns as it is very common in this area. Samples are taken at birth and documented in all files. Here, they deal with blood disorders very efficiently; and it is not included in this study and only errors of metabolism were included.

Personal information like date of birth, sex, area of residence, mother’s age at birth, father’s age, order of birth, birth weight, gestational age on birth, medical history and degree of consanguinity among parents were noted on a standardized pre-tested form.

The principal BD as per the International Classification of Diseases-10 (ICD-10) code was also noted. The children were managed by pediatric services either in Al Ahsa MCH or referred to the specialized King Faisal Specialized Center in Riyad. The identities of children with BD and their parents were de-linked from information on risk factors of BD and only the principal investigator had access to this information [14,15].

The data on live births, children with low birth weights (< 2.5 kg) and the proportion of twin births, to determine plurality, was provided by the Department of Health Information and Statistics. The proportion of preterm babies in general population was 9.4% in a study in Saudi Arabia [16].

The data were analyzed by using Epi Info™ 6 software (Centers for Disease Control and Prevention, USA). After ensuring completion of information, univariate analysis was carried out using the parametric method and the Statistical Package for Social Studies (SPSS, Version 18). Frequencies and incidence of BDs and metabolic errors were calculated per 100 live births. The association between some socio-demographic factors and occurrence of both BDs and inborn metabolic errors, by comparing them with normal cases was estimated using the odds ratio (OR), 95% confidence intervals (CI) and P values (set at (< 0.05). This was used to quantify the risk and denotes to the clinical significance of birth defects and IEM.

## Results

Out of 38,001 live births in the study period, 37,168 were screened and followed until final diagnosis was done. Birth defects (BDs) were found in 426 cases (1.14%) and inborn errors of metabolism, congenital adrenal hyperplasia and hypothyroidism were detected in 63 cases (0.17%).

The incidence of BDs as seen in table 1 was distributed as the diagnosed conditions. The most common birth defects were craniofacial malformations, cardiac, external genitalia and multiple birth defects. They were present in 61, 51, 42 and 66 cases respectively.

Table 2 shows the frequency of the metabolic errors. The 3-methylcrotonyl-CoA carboxylase deficiency (3-MCC) was the most common; followed by biotindase deficiency, hypothyroidism, represented in 13, 12 and 12 cases respectively.

Univariate analysis was shown in tables 3 and 4. Maternal age below 20 years or more than 45 years and paternal age more than 45 years were associated with slight increased risk of BDs; however these risks were not significant. Also, maternal age more than 45 years was associated with slight increased risk of IEM, however this risk was not significant.

Univariate analysis proved that consanguinity, rural residence, and prematurity were associated with significant rise in BDs; with their ORs (CI) are 1.54 (1.24-1.92), 1.29(1.03-1.61) and 1.26(1.02-1.57) respectively. On the other hand, consanguinity and low birth weight were associated with significant rise in metabolic errors; with their ORs (CI) are 1.88(1.04-3.41) and 1.81(1.00-3.29) respectively.

## Discussion

Medical services in the Kingdom have improved tremendously over the past two decades, and health care services are available to the public. Efforts in awareness and management regarding hereditary disorders are currently encouraged. The health services are provided freely by Al-Ahsa Maternity Hospital and a network of 51 primary health care centers. Health insurance is guaranteed freely by the government for all populations. In addition, other facilities in the private sector are provided as in private dispensaries, ARAMCO Petroleum Company Hospital and National Guard hospital.

This study was the first one to be done in Al Ahsa MCH, exploring the birth prevalence of birth defects (BDs) and inborn errors of metabolism (IEM) in live births. Stillbirth and aborted preterm infants were not studied.

In the current study, the most common birth defects were craniofacial malformations, cardiac, external genitalia and multiple birth defects. They were present in 61, 51, 42 and 66 out of 426 cases respectively. The most common metabolic errors included errors in 3-methylcrotonyl-CoA carboxylase (3 –MCC) followed by biotindase and hypothyroidism, represented in 13, 12 and 12 out of the diagnosed 63 cases respectively. Multiple studies discussed the different BDs in Saudi Arabia. One study reported that the incidence of spina bifida in the city of Al-Madinah Al-Munawarah was similar to those reported from the Eastern Province of Saudi Arabia. The consanguinity of parents was a significant risk factor [17]. Another large study was conducted through the Congenital Heart Disease Registry at King Faisal Specialist Hospital in Riyadh. Data indicate that the proportion of cousins in the CHD sample is higher than the proportion in the general population. First-cousin marriage may be a significant risk factor for specific types of congenital heart disease including septal defects, pulmonary stenosis, and pulmonary atresia [18]. El-Mouzan MI reported that a borderline statistical significance was found for major birth defects. The most significant association with all consanguinity was congenital heart disease (CHD) [19].

A prospective study of the antenatal diagnosis of major fetal congenital anomalies conducted in the Women’s Specialized Hospital at King Fahad Medical City from March 2005 to February 2007. Out of 5379 delivered babies, 217 cases of fetal anomalies were diagnosed (27.96/1000).

Genitourinary and cranial anomalies were the commonest; (34.57 per 1000 births). The perinatal mortality rate was 34.9% (65/186), including all cases of intrauterine fetal and neonatal deaths [20].

One large study reported the incidence of inborn errors of metabolism in a cohort of births at the Saudi Aramco medical facilities in the Eastern Province of Saudi Arabia over 25 years from 1983 to 2008. Out of 165 530 Saudi Arabian infants, 248 were diagnosed with an IEM, corresponding to a cumulative incidence of 150 cases per 100 000 live births. Small-molecule disorders were diagnosed in 134/248 patients (54%). Lysosomal storage disorders (LSDs) were the most frequently diagnosed (30%), Organic acid disorders were the second largest IEM group (20%). Methyl malonic aciduria (MMA) constituted the largest subgroup of patients with organic acidurias (14/48 patients) [21].

Many publications reported the relation between extremes of maternal and paternal ages with different birth defects including Down syndrome [22-24].

In the current study, maternal and paternal age more than 45 years were associated with slight increased risk of BDs; however these risks were not significant. Also, maternal age more than 45 years was associated with slight increased risk of IEM; however this risk was not significant. Noninclusion of the stillbirths and abortions may be an affecting factor regarding the calculation of total affected cases and may affect that association.

In the current study, prematurity was associated with significant rise in BDs; ORs (CI) is 1.26 (1.02-1.57). On the other hand, low birth was associated with significant rise in metabolic errors; ORs (CI) 1.81(1.00-3.29). Low birth weight and preterm babies are well known risk factors for BD [25-27]. However, Khandekar and Jaffer found that, low birth weight was negatively associated to BD [24].

In the current study, consanguinity was associated with significant rise in BDs; OR (CI) are 1.54 (1.24-1.92). On the other hand, consanguinity was associated with significant rise in metabolic errors; OR (CI) are 1.88(1.04-3.41).

A myriad of studies discussing the consanguinity in Saudi Arabia were reported. A study was conducted on 3212 Saudi families and found that the consanguinity was variable in different areas with overall rate of 57.7% of the families screened. The most frequent were first cousin marriages (28-4%) [28]. Consanguineous marriages were reported to be as high as 60% and this has provided a background in which these genetic diseases abound [29,30].

A cross-sectional study was done using multistage random probability sampling of Saudi population taken from the 13 regions of the Kingdom. The overall prevalence of consanguinity in the Kingdom of Saudi Arabia is high; 56% with the first-degree cousin (33.6%) being more common than all other relations (22.4%). The overall prevalence was significantly more common in rural (59.5%) than in urban settlements (54.7%) [31]. It was reported that the high incidence of pediatric congenital or genetically-determined disorders in Arabian Peninsula resulted from the heavy consanguineous marriages and the tribal nature of the marriages, both of which led to the preservation of rare mutations kept in a genetically homogenous population [32]. Al-Aqeelv et al. reported that Middle East countries including Saudi Arabia had first cousin marriages account for 60 - 70% of all marriages, leading to uniquely common disorders which are either rare by Western standards or are unknown [33].

In the current study, univariate analysis proved that rural residence was associated with significant rise in BDs; ORs (CI) 1.29 (1.03-1.61). The rural conditions may encourage more consanguineous marriage or may increase the exposure to waste of the different insecticides and pesticides. Migration to urban areas and the growth of the nuclear family has also been postulated to dilute the role of consanguinity and that could be the reason for the observed association of consanguinity with BD in our study [34].

The current study has some limitations which should be considered before making extrapolation. It was a retrospective with multiple areas of uncontrolled bias either in enrollment or data finding. We didn’t include abortions and stillbirths, which may decrease the magnitude of the problem limiting the number of diagnosed cases. Absence of genetic maps limited our ability to trace all genetic errors in certain families. Finally, the different patterns and percentages of consanguinity decrease the extent of national generalization of results, but it does add a piece of information to what is currently present.

## Conclusions

First cousins consanguinity represented the most significant risk factor for birth defects and inborn errors of metabolism. High degree of inbreeding, consanguinity may exacerbate underlying recessive genetic risk factors. We shouldn’t neglect the positive aspects assumed to be in these marriages. They are claimed to be associated with more socially stable and economically beneficial, family fortune, with preserved extended family tribe. Putting all cons and pros together, should help in the awareness about early detection and lines of management of consanguinity related disorders.

## Competing interests

The authors read the manuscript and approved it. No conflict of interest was present. No source of funding.

## Authors’ contribution

The authors jointly conceived and designed this study, collected data, analyzed, and interpreted the data. They also took part in drafting the article and revising it critically for important intellectual content; and finally approved this version to be published. AWH: Literature search, review of the

results, data interpretation and manuscript critical review. BMH: conception and design, literature search-data analysis and interpretation, manuscript editing and final review. MS: Design, literature search, manuscript drafting, and review. HI: Data acquisition and manuscript review

## Acknowledgments

The authors would like to thank the MCH filing unit for nice documentation and help. All thanks are to the Department of Pediatrics, Neonatology section for their help and efforts in serving the local community and allowing collecting the data. Also, thanks to the efforts of the students, Talal Al Ma khlafy, Tarek Al Yahia, we could finalize our data collection accurately.

## Figures and Tables

**Table 1:** Number and percentage of confirmed birth defects in a population of children born with birth defects from 2006 to 2009 in the neonatology section, Maternity Hospital, Al Ahsa, Eastern Region, Saudi Arabia

**No of fully explored live birth cases**	**Confirmed Cases**	**Total cases of birth defects**		
		**Type**	**No**	%
37168	426 (1.14%)	Craniofacial	61	14.32
		NTDs	22	5.16
		Cardiac	51	11.97
		CDH	16	3.76
		Abd massess	37	8.69
		Abdominal wall	11	2.58
		GIT	23	5.40
		Ext Genitalia	42	9.86
		CHD	26	6.10
		Skletal	17	3.99
		Trisomy	26	6.10
		Hydrops	12	2.82
		Others	16	3.76
		Multiple	66	15.49

**Table 2:** Number and percentage of inborn errors of metabolism in a population of children born with inborn errors of metabolism from 2006 to 2009 in the neonatology section, Maternity Hospital, Al Ahsa, Eastern Region, Saudi Arabia

		**Total confirmed cases of metabolic errors**		
**No of Screened Live births**	**Confirmed Cases**	**Type**	**No**	**%**
		3-methylcrotonyl-CoA carboxylase (3– MCC)	13	20.63
		Biotinidaze deficiency	12	19.05
		Medium Chain acyl Coenzyme A Dehydrogenase (MCAD)	5	7.94
		Glutaric Aciduria	1	1.59
		Glutaric Academia type 2	4	6.35
		Probionic Acidemia	1	1.59
		Homocysteinuria	2	3.17
		Primary Carnitin deficiency	1	1.59
37168	63 (0.17%)			
		Nonketotic hyperglycinaemia (NKH)	2	3.17
		Galactosemia	2	3.17
		Phenyl Ketonuria (PKU)	1	1.59
		Methylmalonic acidaemia (MMA)	2	3.17
		Multiple corboxylase deficiency	1	1.59
		Citrulinemia	1	1.59
		Hypothyroidism	12	19.05
		Congenital adrenal hyperplasia (CAH)	3	4.76

**Table 3:** Univariate analysis of risk factors of birth defects in a population of children born with birth defects from 2006 to 2009 in the neonatology section, Maternity Hospital, Al Ahsa, Eastern Region, Saudi Arabia

**Parameters**	**Control group Live birth N = 1768, No (%)**	**Bird defects group N = 426, No (%)**	**Odds ratio (95% confidence intervals)**
**Maternal age groups**			
≥20-45	924 (52.26)	222 (52.11)	Reference
<20	366 (20.70)	96 (22.53)	1.09 (0.83-1.44)
>45	478 (27.04)	138 (32.39)	1.2 (0.94-1.54)
**Father age groups**			
≤45	914 (51.69)	242 (56.81)	Reference
<45	854 (48.30)	184 (43.19)	0.81 (0.65-1.01)
**Consanguinity**			
Present	878(49.66)	246(57.74)	1.54 (1.24-1.92)*
Absent	990 (55.99)	180 (42.25)	Reference
**Residence**			
Urban	764 (43.21)	158 (37.08)	Reference
Rural / Hagar	1004 (56.78)	268 (62.91)	1.29(1.03-1.61)*
**Birth weight**			
≥2.5 kg	967 (54.69)	236 (55.39)	1.03(0.83-1.28)
<2.5 kg	801 (45.30)	190 (44.60)	Reference
**Gestational age groups**			
≤37 w	860 (48.64)	232 (54.46)	1.26(1.02-1.57)*
≥37 w	908 (51.36)	194 (45.53)	Reference

**Table 4:** Univariate analysis of risk factors of inborn errors of metabolism (IEM) in a population of children born with inborn errors of metabolism from 2006 to 2009 in the neonatology section, Maternity Hospital, Al Ahsa, Eastern Region, Saudi Arabia

**Parameters**	**Control group Live birth N = 250, No (%)**	**Metabolic errors group N = 63, No (%)**	**Odds ratio (95% confidence intervals)**
**Maternal age groups**			
≥20-45	114 (45.6)	30 (47.6)	Reference
< 20	67 (26.8)	14 (22.2)	0.79 (0.37-1.69)
<45	69 (27.6)	19 (30.16)	1.05 (0.52-2.01)
**Father age groups**			
≤45	117 (46.8)	34 (54.0)	Reference
>45	133 (53.2)	29 (46.0)	0.75 (0.42-1.35)
**Consanguinity**			
Present	96 (38.4)	34 (54.0)	1.88(1.04-3.41)
Absent	154 (61.6)	29 (46.0)	Reference
**Residence**			
Urban	112 (44.8)	37(58.7)	1.75 (0.97-3.19)
Rural / Hagar	138 (55.2)	26 (41.37)	Reference
**Birth weight**			
≥2.5 kg	148 (59.2)	28 (44.44)	Reference
< 2.5 kg	102 (40.8)	35 (55.55)	1.81(1.00-3.29)
**Gestational age groups**			
≤37 w	93 (37.2)	28 (44.44)	1.35(0.74-2.45)
≥37 w	157 (62.8)	35 (55.55)	Reference
